# The deficient cue monitoring and the facilitating effect of prosocial intention on prospective memory in patients with schizophrenia spectrum disorders

**DOI:** 10.1038/s41537-023-00363-y

**Published:** 2023-05-23

**Authors:** Dong-Yang Chen, Qi Wang, Ning-Bo Yang, Xiao-Jing Qin, Hang Li, Wen-Peng Hou, Yu-Shen Ding, Wei-Wei Hou, Ya Wang, Fu-Chun Zhou, Chuan-Yue Wang

**Affiliations:** 1 The National Clinical Research Center for Mental Disorders & Beijing Key Laboratory of Mental Disorders Beijing Anding Hospital & the Advanced Innovation Center for Human Brain Protection, Capital Medical University, School of Mental Health, Beijing, China; 2 Beijing Fengtai Mental Health Center, Beijing, China; 3 First Affiliated Hospital of Henan University of Science and Technology, Luoyang, China; 4 Neuropsychology and Applied Cognitive Neuroscience Lab, CAS Key Laboratory of Mental Health, Institute of Psychology, Beijing, China; 5 Guangxi Medical University, Nanning, China

**Keywords:** Psychosis, Human behaviour

## Abstract

The study aimed to investigate the cognitive processing of prospective memory (PM) in patients with schizophrenia spectrum disorders (SSDs) by using an eye-tracking paradigm. In addition, the facilitating effects of prosocial intention (the desire to help others) on PM in SSDs were also examined. In phase 1, 26 patients (group1) and 25 healthy controls (HCs) were compared in an eye-tracking PM paradigm in terms of the PM accuracy and eye-tracking indices. In phase 2, 21 more patients (group2) were recruited, and a prosocial intention was introduced in the eye-tracking PM paradigm. Their PM accuracy and eye-tracking indices were compared with those in group1. The PM cue monitoring was indicated by the total fixation counts and fixation time on distractor words. In phase 1, group1 showed lower PM accuracy, fewer fixation counts and less fixation time on distractor words than HCs. In phase 2, group2 (with prosocial intention) performed significantly better than group1 (with typical instruction) on both PM accuracy and fixation time on distractor words. In both groups of SSDs, the PM accuracy was significantly correlated with both the fixation counts and the fixation time of distractor words. After controlling for the cue monitoring indices, the difference in PM accuracy remained significant between group1 and HCs but disappeared between group1 and group2. The cue monitoring deficit contributes to the PM impairment in SSDs. The facilitating effect of prosocial intention disappears after the control of cue monitoring, also indicating its critical role in PM.

## Introduction

Cognitive impairment is one of the core symptoms in schizophrenia^1,2^. Among cognitive dimensions, memory is always significantly impaired in different stages of the disease^3^. It has been reported that 50–80% of all everyday memory complaints could be attributed to the failure to remember to perform something in the future, which is defined as prospective memory (PM)^4,5^. Previous studies found that schizophrenia patients with PM deficit exhibited a high level of functional disability^6^. Prefrontal cortex, in particular BA10 and related network, has been consistently observed to be involved in functional imaging studies of PM^7^. However, the underlying neural mechanisms of the PM deficits in patients with schizophrenia are not fully unraveled. With the limited understanding of the mechanisms of PM deficits, the interventions for improving PM performance in patients with schizophrenia is still under development. In view of the importance of PM in our daily life and its negative impacts on the functional outcomes of patients with schizophrenia, it is an urgent matter to resolve this problem.

PM is usually divided into event-based PM and time-based PM. Event-based PM refers to the ability to remember to execute an intention when an event/cue appears^8^. Time-based PM involves self-initiation and execution of the previously formed intention at a specific moment. In the process of PM, the person has to keep in mind the previously formed intention while participating in other ongoing activities during the period of delay^3^. The neurocognitive process underlying PM involve four stages, namely intention formation, intention retention, intention initiation and intention execution^9^. Intention initiation consists of two important components, namely, cue detection and intention retrieval. Cue detection refers to the recognition of cue event signaling that an intended action should be performed, while intention retrieval means the retrieval of an intention from long-term memory following the recognition of a prospective cue^10,11^. Theoretical hypotheses (e.g., strategic monitoring and spontaneous retrieval) have been proposed to explain how an individual accomplishes a PM task^12,13^. Strategic monitoring is typically accompanied by a search experience and spontaneous retrieval is typically accompanied by a pop-up experience^14^.

Cue focality (focal vs. non-focal) is an important factor affecting PM processing^15,16^. A PM cue is labeled focal when it is stimulated by processing of the ongoing task stimuli (e.g., talking to a friend about football is likely to stimulate remembering to watch the FIFA world cup). Conversely, the feature of a non-focal cue is irrelevant to the processing of ongoing stimuli (e.g., talking to a friend about football is less likely to trigger remembering to return a book at the library)^17,18^. As interpreted in multiprocessing theory^19^, the intention retrieval depends more on strategic monitoring in non-focal tasks, which is closely associated with the activity of left anterior prefrontal cortex and anterior cingulate cortex. In contrast, the PM intention is spontaneously retrieved in the less demanding focal tasks, which reflects the function of cerebellum and ventral parietal regions. These findings suggests that a top-down processes (monitoring) are more involved in non-focal tasks, whereas the focal tasks are primarily mediated by bottom-up processes (spontaneous retrieval)^15^. Cue focality has not yet been systematically investigated in patients with schizophrenia. Nevertheless, the effect of cue focality on PM performance has been examined in college students with high schizotypal traits (HSTs) and or low schizotypal traits (LSTs) respectively^20^. The results indicated lower PM accuracy and less PM cue monitoring in individuals with HSTs compared with LSTs. However, when the data were re-analyzed separately, the difference between HSTs and LSTs in PM accuracy only exits in the non-focal paradigm^20^.

A growing body of evidence indicated that patients with chronic schizophrenia^21,22^ and first-episode schizophrenia^23,24^ both exhibited PM deficits. However, the information processing mechanisms of impaired PM in schizophrenia remains unclear. Several studies have examined the specific components of impaired PM in schizophrenia, and cue detection has been found to be significantly impaired in schizophrenia even after controlling for working memory^25^. Unfortunately, these studies relied primarily on subjective recall and behavioral experiments, and PM tasks were presented by using a single stimulus^25–27^.

Recently, several studies have used eye-tracking to investigate PM^28–31^. The eye-tracking research of PM usually utilizes visual search task with multiple stimuli presentation thus provide more insight into the processing of PM^32–34^. It has been considered that event-based PM has wider implications in everyday activities^35^. Event-based PM paradigm also allows to examine cue processing, which plays a key role in modulating PM performance^25^. In the eye-tracking paradigm, identifying PM cues in multiple stimuli closely resembles the situation in real world^28,36,37^. For example, the total fixation counts of distractor (stimuli other than targets and PM cues in visual searching tasks) sensitively reflected the individual’s strategic monitoring ability of PM cues^32,38^.

Nevertheless, majority of these studies were conducted in healthy participants^28–31^, only a few were conducted in subclinical or clinical populations such as in HSTs (individuals with high-risk traits for schizophrenia)^20^, and patients with depression^36^. It seems that the impaired ability of strategic monitoring is a significant predictor to PM impairment in patients with depression^36^ and HSTs^20^. It is still unknown whether this conclusion can be generalized to other clinical population, such as schizophrenia. Therefore, one aim of the present study was to address this issue.

In PM studies, researchers have tended to focus on the neurocognitive aspects of PM, while ignoring the basic social and motivational aspects of this activity^39^. Prosocial prospective memory (PSPM) refers to the ability to complete the PM task driven by prosocial intention^40^. Prosocial intention is the desire of individuals to develop and demonstrate prosocial behavior, which is a sign of people’s willingness to help others^41^. In previous PSPM studies, pro-social intentions were introduced by instructions before the experiment. The experimenter usually asked the participants to do them a favor by performing the PM task (adding social importance). It has been found that prosocial intention significantly facilitated the PM performance in healthy volunteers^40,42,43^, but not in cognitively impaired non-demented individuals^44^. So far, the facilitating effect of prosocial intention has not yet been examined in patients with schizophrenia. As schizophrenia patients are impaired in social cognition and exhibit less prosocial behavior^45^, it is unclear whether the facilitating effect of prosocial intention still exist. If so, it is interesting to find out the underlying cognitive processing mechanisms by using an eye-tracking paradigm.

The hypotheses of the present study included the following: (1) the impaired strategy monitoring is a key cognitive processing mechanism of PM impairment in patients with schizophrenia spectrum disorders (SSDs) in a non-focal event-based PM paradigm by using eye tracking techniques; (2) the prosocial intention has a facilitating effect on PM performance in SSDs, which can be explained by the underlying deficient cue monitoring ability.

## Method

### Participants

This study was conducted in the Beijing Anding hospital, Capital Medical University, a tertiary psychiatric hospital with 800 beds. In phase 1, 26 patients (group1) and 25 HCs were compared in a non-focal event-based PM eye-tracking paradigm, in terms of their PM performance and eye movements. In phase 2, the prosocial intention was introduced by instructions in 21 patients (group2). Consequently, 47 SSDs from the outpatient and inpatient departments were enrolled in the study.

All patients met the diagnostic criteria for schizophrenia and other psychotic disorders (e.g., schizophreniform disorder, schizoaffective disorders) according to the Diagnostic and Statistical Manual of Mental Disorders, Fifth Edition (DSM-5)^46^. The diagnosis was confirmed by a research psychiatrist using the Mini-International Neuropsychiatric Interview version 7.0.2 (MINI 7.0.2)^47^. Other inclusion criteria included (1) at least 9 years of education; (2) age between 18 and 40 years old; (3) right-handed; (4) clinically stable and the medication plan unchanged over the past 3 months. Furthermore, exclusion criteria were the following: (1) IQ 80 or below (measured by the short forms of Wechsler Adult Intelligence Scale (WAIS-RC); (2) received electric convulsive therapy (ECT) or any neuromodulation therapy (e.g., transcranial magnetic stimulation) within past 6 months; (3) current or past substance abuse, severe neurological diseases or other medical conditions that may impact cognition.

In addition, 25 health individuals (HCs) were recruited from nearby communities who were matched with the patients in term of age, sex, and education. They also went through MINI screening to exclude any diagnosable mental disorders.

This study was approved by the Institutional Review Board of the Beijing Anding Hospital. Written informed consent was obtained from all participants before they enter the study.

### PM assessment

**Fig. 1:**
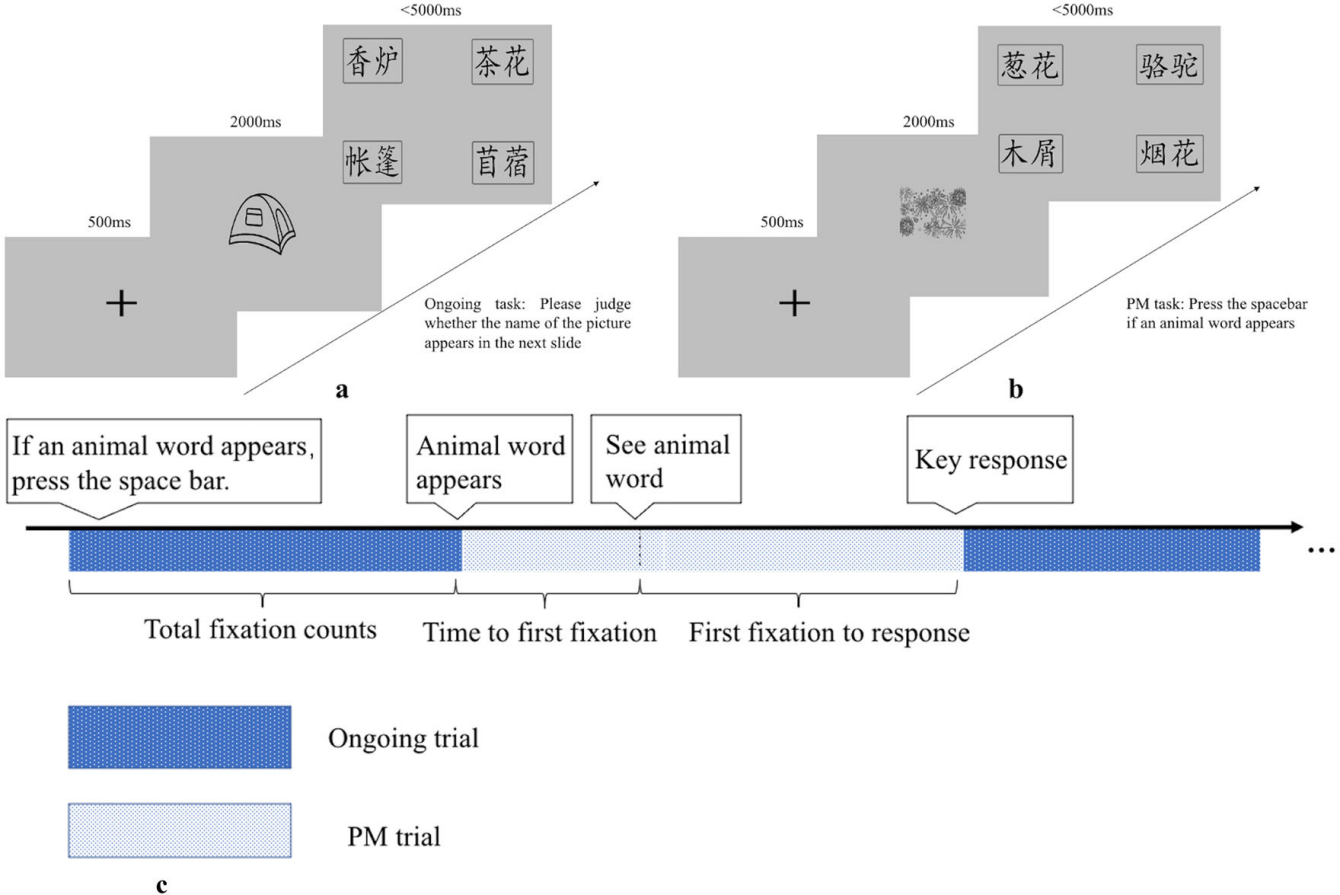
Illustration of the prospective memory experiment. **a** In an ongoing trial, participants are asked to identify the object in the first slide and then search the word on the second slide that describes the object. **b** In a nonfocal PM trial, participants were supposed to find any animal words while doing the ongoing task. **c** The experiment flow. PM = prospective memory.

The experiment flow was shown in Fig. 1. It is a dual-task non-focal event-based PM paradigm. The experiment includes a practice session (only including ongoing tasks) before the formal PM session. For the ongoing tasks, stimuli included 330 figures (simple line-drawing) and 1320 nouns selected from Consortium Chinese Linguistic Data (2003). In each trial of the task, a figure of object was shown in the center of the screen with an 8.5° × 8.5° visual angle for 2000 ms. Then four words (may include a target word, may include a PM cue, and distractor words) were presented at the four corners of the screen. These words were spaced ~5.7° horizontally and 3.3° vertically each with a visual angle of 4° × 2°. The target words and PM cue words evenly appeared at each quadrant of the screen throughout the task.

Participants were required to identify the object in the first slide and then search on the second slide for the word that exactly matched the object. They were instructed to press the “j” key when a target word was detected; otherwise, they would press the “f” key. The words slide disappeared automatically as soon as the response was made or after 5 s of no response. In phase 1, the standard PM session consisted of 2 blocks of non-focal PM task. The participants were asked to press the “spacebar” if they saw any animal words while doing the ongoing task, and this was defined as the PM task. There were 6 PM trials distributed among 74 ongoing trials in each block. Among the 80 trials, 40 of them contained the target word (name of the previously seen object, such as “elephant”). There was 1 min break between the two blocks.

In phase 2, in order to make the prospective task socially relevant, the participants in the prosocial group were given the following instruction prior to the experiment, “This task is developed as cognitive training program to improve patients’ cognitive function. Your accuracy will be used to judge whether this cognitive training program is effective. In order to benefit more patients in the future, please try your best to make correct responses”.

### Clinical assessment

The Mini International Neuropsychiatric Interview (MINI) was designed as a brief structured diagnostic interview for the major psychiatric disorders, which has been widely used in both clinical and research settings. Validation and reliability studies have shown that the MINI has similar psychometric properties to other structured diagnostic instruments but can be administered in a much shorter period of time^47^. The patients’ psychopathology was evaluated with the Positive and Negative Syndrome Scale (PANSS)^48^ by their treating psychiatrists who have been trained to use the scale with good inter-rater reliability.

### Apparatus

A CRT monitor was used to present stimuli. The resolution of the screen was 1024 by 768 pixels, and the refresh rate was 60 Hz. The distance between the participants’ eyes to the monitor was ~60 cm. Eye tracking data were recorded binocularly at 1000 Hz using an EyeLink1000 eye tracker (SR Research, Ottawa, Ontario, Canada). All participants had to complete a 9-point calibration before the experiment. If an error in any fixation point exceeded 1° or if the average error for all points was more than 0.5°, the calibration was repeated.

### Statistical analyses

The analyses of behavior data involved the accuracy and response time of PM trials (PM-ACC, PM-RT) and ongoing trials (OT-ACC, OT-RT). Data Viewer 3.2 was used to extract eye-tracking data. Blink artefacts and fixation (gaze) below 80 ms were eliminated. Eye-movement data was analyzed based on the regions of interest (ROI), which was 238 × 144 pixels in resolution with a visual angle of ~5° × 3°. The following eye movement indices were used in analyses: (1) the total fixation count for distractor words, which is the total numbers of gazes on distractor words in the ongoing trials; (2) the total fixation time for distractor words, which is the total time of all fixations spent on the distractors in the ongoing trials; (3) the time to first fixation refers to the interval between the onset of word stimuli and the first fixation within the ROI of PM cues; (4) the time from first fixation to response means the interval between the occurrence of the first gaze within the ROI of PM cues and the response. Among these indices, the total fixation count for distractors and the total fixation time for distractors were used to indicate the process of cue monitoring. The time to first fixation reflects the individual’s alertness to stimuli and the time from first fixation to responses indicates the time spent on intention retrieval and execution.

Statistical analyses were conducted using SPSS 26.0 software package. One-sample Kolmogorov Smirnov tests were used to check the normality of distributions for the continuous variables. The group comparisons regarding continuous variables were analyzed by using *T*-test for normally distributed data or Mann–Whitney U-test for skewed data. For categorical variables, chi-square test was for group comparisons. Pearson correlation or spearman’s rank correlation was used to examine the relationships between PM-ACC and eye movement indices. Two-tailed tests were used, and the significant level was set as *P* < 0.05.

In phase 1, an Analysis of covariance (ANCOVA) was conducted to determine whether the group difference in PM-ACC (SDDs vs. HCs) was totally attributed to the variance change in cue detection. In phase 2, another ANCOVA was carried out to examine the extent to which cue detection difference accounts for the PM-ACC difference between standard and prosocial paradigms.

## Results

### Phase 1

**Fig. 2:**
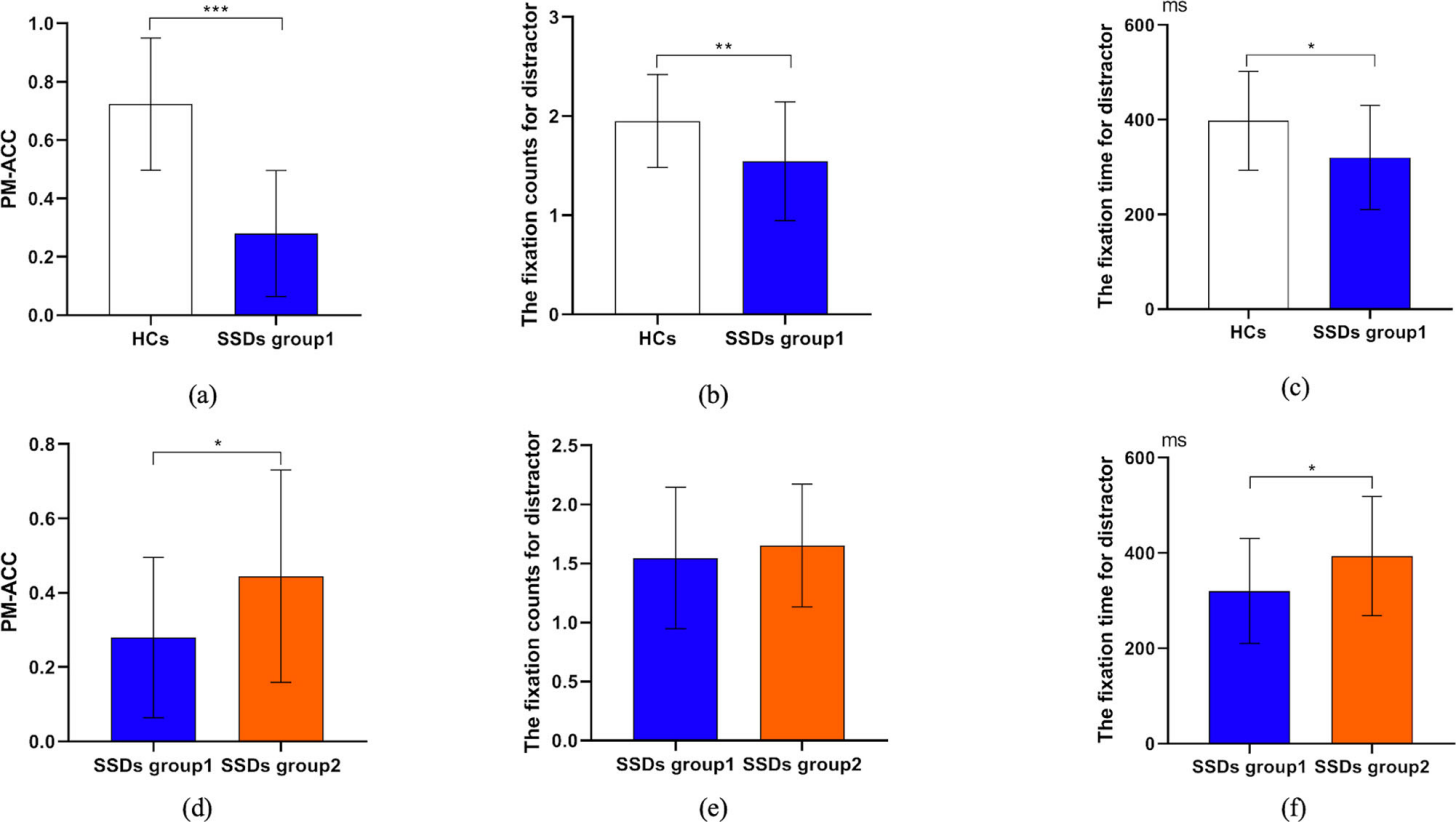
Behavioral and eye tracking measures of PM in each group in phase 1 and phase 2. SSDs=Patients with schizophrenia spectrum disorders, SDDs group1=SDD patients with typical instructions, SDDs group2=SDDs patients with prosocial instructions, HCs=Healthy controls, PM-ACC=the accuracy of PM trials, Total fixation counts=total fixation counts for distractor words, Total fixation time=total fixation duration for distractor words. **a** The comparison of PM-ACC between the HCs and SSDs group1. **b** The comparison of the fixation counts for distractor between HCs and SSDs group1. **c** The comparison of the fixation time for distractor between the HCs and SSDs group1. **d** The comparison of PM-ACC between the SSDs group1 and SSDs group2. **e** The comparison of the fixation counts for distractor between the SSDs group1 and SSDs group2. **f** The comparison of the fixation time for distractor between the SSDs group1 and SSDs group2.

Table 1 shows the basic demographic variables, the mean performance on the PM and ongoing tasks, and eye movement data in the group1 and HCs, as well as the clinical characteristics for group1. There were no significant differences in age, gender, and education level between the patients and HCs. The means of OT-ACC were above 0.85 in both groups, indicating adequate engagement of participants in both groups. The patients’ PM-ACC, OT-ACC, OT-RT, total fixation count and total fixation time for distractor words were significantly lower than those of the HCs (Fig. 2).

**Table 1:** Group comparisons with respect to demographic, the performance of prospective memory and ongoing tasks, eye tracking data and psychopathology in phase 1 and phase 2.

	SSDs				HCs(*n* = 25)		Statistics					
	Group1 (*n* = 26)		Group2 (*n* = 21)				Group1 vs. HCs			Group1 vs. Group2		
	N/Mean	Percent/SD	N/Mean	Percent/SD	N/Mean	Percent/SD	X^2^ /t/z	df	*p* value	X^2^ /t/z	df	*P* value
Gender(men)	14	53.8%	10	47.6%	14	56%	0.024	1	0.877	0.18	1	0.671
Age(years)	29.31	6.41	27.43	6.45	26.52	4.47	1.81	49	0.079	0.98	45	0.324
Education(years)	14.62	2.59	14.52	2.64	16.40	2.81	−1.90	–^a^	0.058	0.20	–^a^	0.845
Course of illness(years)	6.73	5.39	5.73	4.78	–	–	–	–	–	0.37	–^a^	0.714
Diagnosis of schizophrenia	24	92.3%	17	80.9%	–	–	–	–	–	1.35	1	0.246
PANSStot	60	11.58	54.09	12.32	–	–	–	–	–	1.69	45	0.098
PANSSpos	12.82	4.68	12.15	4.28	–	–	–	–	–	0.50	45	0.619
PANSSneg	17.80	6.49	14.95	5.07	–	–	–	–	–	1.65	45	0.106
PM-ACC	0.28	0.22	0.44	0.29	0.72	0.23	−5.16	–^a^	<0.001	−2.27	45	0.028
PM-RT (ms)	2056.72	539.69	2342.55	478.13	2081.04	504.40	−0.17	49	0.869	−1.90	45	0.064
OT-ACC	0.87	0.10	0.89	0.10	0.97	0.02	−4.98	–^a^	<0.001	−0.88	–^a^	0.379
OT-RT (ms)	1862.87	538.06	2204.06	403.60	2280.82	493.48	−2.89	49	0.006	−2.41	45	0.020
Total fixation counts	1.55	0.60	1.65	0.52	1.95	0.47	−2.68	–^a^	0.007	−0.75	–^a^	0.454
Total fixation duration (ms)	320.44	109.92	393.45	125.45	397.62	104.28	−2.57	49	0.013	−2.13	45	0.039
Time to first fixation (ms)	740.50	228.24	799.80	225.08	639.35	156.94	1.84	49	0.072	−0.89	45	0.378
First fixation to response (ms)	1320.16	468.61	1542.74	435.93	1441.69	488.10	−0.91	49	0.369	−1.67	45	0.102

*SSDs* Patients with schizophrenia spectrum disorders, *Group1* SDD patients with typical instructions, *Group2* SDDs patients with prosocial instructions, *HCs* Healthy controls, *PANSS* Positive and Negative Syndrome Scale, *PM-ACC* the accuracy of PM trials, *PM-RT* the response time of PM trials, *OT-ACC* the accuracy of Ongoing trials, *OT-RT* the response time of Ongoing trials, *Total fixation counts* total fixation counts for distractor words, *Total fixation duration* total fixation duration for distractor words, *Time to first fixation* the interval between the onset of word stimuli and the first fixation within the ROI of PM cues, *First fixation to response* the interval between the occurrence of the first gaze within the ROI of PM cues and the response, *PANSSpos* Positive subscale score of the Positive and Negative Syndrome Scale, *PANSSneg* Negative subscale score of the Positive and Negative Syndrome Scale, *PANSStot* Total score of the Positive and Negative Syndrome Scale.

^a^Mann–Whitney U-test.

In order to examine the association between eye movement indices and PM performance, a series of association analyses was conducted in the whole sample. Table 2 presented that PM-ACC was significantly associated with the total fixation count and total fixation time for distractor words.

**Table 2:** Correlation between the eye movement indices and the accuracy of PM tasks in phase 1 and phase 2 respectively.

Measures	Phase 1 (*n* = 51)		Phase 2 (*n* = 47)	
	*r*	*p* value	*r*	*p* value
Total fixation counts	0.51^a^	<0.001	0.67^a^	<0.001
Total fixation time	0.51^a^	<0.001	0.62	<0.001
Time to first fixation	−0.29^a^	0.041	0.05^a^	0.721
First fixation to response	0.21^a^	0.138	0.41	0.004

*Total fixation counts* total fixation counts for distractor words, *Total fixation time* total fixation duration for distractor words, *Time to first fixation* the interval between the onset of word stimuli and the first fixation within the ROI of PM cues, *First fixation to response* the interval between the occurrence of the first gaze within the ROI of PM cues and the response.

^a^Spearman’s rank correlation.

An ANCOVA was conducted to determine whether the group difference in PM-ACC (SDDs vs. HCs) was totally due to cue detection differences. After controlling for total fixation count and total fixation time for distractor words, the difference between the two groups with respect to PM-ACC remained (*F*_*(1, 47)*_ = *37.5, partial η*^*2*^ = *0.44, P* < *0.001*).

### Phase 2

The basic demographic variables, the mean performance on the PM and ongoing tasks, and eye movement data and clinical characteristics in the group2 and group1 were shown in Table 1. There were no significant differences in age, gender, education level and psychopathology between the two groups of patients. The means of OT-ACC were above 0.85 in both groups, suggesting adequate engagement of participants in both groups. Group comparisons revealed that patients under prosocial condition performed significantly better than those under standard condition in terms of PM-ACC, OT-RT, and total fixation time for distractor words (Fig. 1).

Correlation analyses was conducted to examine the association eye movement indices and PM performance in the whole sample. As shown in Table 2, PM-ACC was significantly associated with the total fixation count and total fixation time for distractor words, and the time from first fixation to response.

In order to examine the extent to which the variance changes of PM-ACC were due to the difference in cue detection between standard and prosocial paradigms, an ANCOVA was conducted. After controlling for the total fixation time for distractor words, the grouping factor was no longer a predictor for the difference in PM-ACC (*F*_*(1, 44)*_ = *1.42, P* = *0.240*). The total fixation time for distractor words was the only significant contributor for the variance of PM-ACC (*F*_*(1, 44)*_ = *22.61, partial η*^*2*^ = *0.34, P* < *0.001*).

## Discussion

In the present study, we examined PM performance in SSDs using a standard and a prosocial non-focal PM eye-tracking paradigm in phase 1 and phase 2 respectively. We found that (1) SSDs exhibited lower PM-ACC and less cue monitoring behavior compared to HCs; PM-ACC was significantly correlated with cue monitoring indices; (2) Prosocial intention significantly improved PM-ACC and cue monitoring in SSDs. Both of the hypotheses were confirmed.

In phase 1, we found that PM-ACC was significantly lower in SSDs than in HCs, which is in line with most previous PM studies in SSDs^3,49^. This proved that the PM of patients with schizophrenia spectrum disorders is impaired on the behavioral level, and also validated the non-focal PM paradigm in SSDs.

Several studies have investigated the underlying cognitive processes of PM impairment in schizophrenia, and cue detection and intention retrieval were considered as the critical impaired phases of PM^25,27,50^. However, the limitations of these studies include the indirect measures for the cognitive processing of PM and the focus limited on the period after PM cue presentation. As in previous reports^20,28^, we used the total fixation counts and the total fixation time for distractor words to directly reflect the effort and time put in cue monitoring in the task environment. Similar to the results in individuals with high schizotypal traits^20^, eye-tracking results also showed that the total fixation counts and total fixation time on distractor words in SSDs were lower than HCs. As outlined in the multiprocess theory, non-focal PM tasks demand high levels of controlled attentional resources^19^, These eye-tracking indices reflect the deficient PM cue monitoring in SSDs, which may be explained by patients’ reduced attention resources^51^. According to the multiprocess theory, the top-down (strategic monitoring) and the bottom-up (spontaneous retrieval) processes are interconnected and dynamically interact to support prospective memory^12,13^. In the current study, the difference of PM-ACC is still present between SSDs and HCs after controlling the cue monitoring, suggesting the pathological process of schizophrenia has also disrupted the “bottom-up” cognitive processes, which has been already reported previously^52^.

In phase 2, we examined the effect of prosocial intention on PM performance in SSDs and its underlying mechanisms. It seems that prosocial intention has the potential to improve PM performance of SSDs. This is consistent with findings from previous studies that applied prosocial intention to healthy individuals^40,42,53,54^. These findings suggest that prosocial intention can be used as an effective strategy to help SSDs realize delayed intentions. The results also showed that SSDs under prosocial condition had a significantly longer fixation time on distractor words than those did under the standard condition. Correlation analyses also confirmed the significant association between PM-ACC and cue monitoring indices. Therefore, it is likely that prosocial intention improved PM performance of SSDs by increasing their cue monitoring. After controlling for the total fixation time for distractor words, the group difference regarding PM-ACC disappeared, which further proved the critical role of cue monitoring in prosocial facilitation.

Several lines of evidence have indicated that the function of inhibitory control was impaired in patients with schizophrenia^55–57^. Prefrontal areas, ACC in particular, have been implicated in the process of monitoring^58^. It indeed takes certain amount of time for the neural networks to loop signals from the ACC to frontal structures so as to engage in monitoring^59,60^. Previous studies demonstrated that an immature inhibitory control system may not provide enough time for these signals to be strengthened and passed on^61^. In such case, additional time is needed for these individuals to process information and conduct accurate monitoring^62,63^. One explanation would be that prosocial intention attached social importance of the PM task thus changed the cognitive resource allocation. The eye-movement results revealed the total fixation time of the distractor words were increased in SDDs under prosocial condition compared to those under standard condition, indicating more time and effort was put in cue monitoring in patients with prosocial intention. Besides, there was no significant differences for the time to first fixation or the time to response between groups, which means the increased total fixation time in group2 was not caused by general slowing.

To our knowledge, this is the first study to investigate PM impairments in SSDs by using an eye-tracking paradigm, which gave us more insights into the cognitive processing deficits in this population. Besides, the facilitating effects of prosocial intention was confirmed, and the underlying cognitive mechanisms were revealed by the eye-tracking paradigm. These are the strengths of the study. However, the results should be interpreted with caution due to the following potential limitations. First, the sample size was relatively small, though the sociodemographic variables and psychopathology were matched between groups. ANCOVA showed the final model accounted for 58.2% (adjusted *R*^2^ = 0.582) of the variance changes of PM-ACC in phase 1, and only 38.0% in phase 2 (adjusted *R*^2^ = 0.38). A larger sample size would allow us include more influential factors in the analyses. Second, PM is a higher order complex cognitive function which is subject to the individual’s basic neurocognitive functions, such as working memory, retrospective memory, cognitive flexibility, and attention^9,10,64,65^. These cognitive domains are also impaired in SSDs but not controlled in the present study. Third, due to the cross-sectional design, the causality of relationships between intervention with prosocial intention and PM performance could not be explored. Finally, there are other cognitive possess that may contribute to PM-ACC group difference (SSDs vs. HCs; prosocial vs. standard paradigm) that we did not explore, such as intention formation. In future studies, modified eye-tracking paradigms are needed to cover all the four stages of cognitive processing of PM.

In conclusion, the major finding of this study is that deficient cue monitoring contributed to PM impairment in SSDs, as revealed by the eye-tracking PM paradigm. The prosocial intention significantly improved PM accuracy in SSDs, which could be explained by an enhancement of cue monitoring. The findings shed light on the underlying cognitive mechanisms of PM, and also identified cue monitoring as a key target for intervention. Moreover, the validated eye-tracking PM paradigm would lend support to the precise evaluation of therapeutic efficacy for PM deficits in the future.
